# Supplementary material for: IL-13/IL-13Rα2 axis promotes proliferation of angiosarcoma cells
Source: Sci Rep. 2025 Aug 25;15:31236. doi: 10.1038/s41598-025-15933-6 (PMC12379233; doi:10.1038/s41598-025-15933-6)
Supplement: Supplementary file 1 — Supplementary Information 1. [file 41598_2025_15933_MOESM1_ESM.pdf]

Supplementary information

Supplementary Figure 1

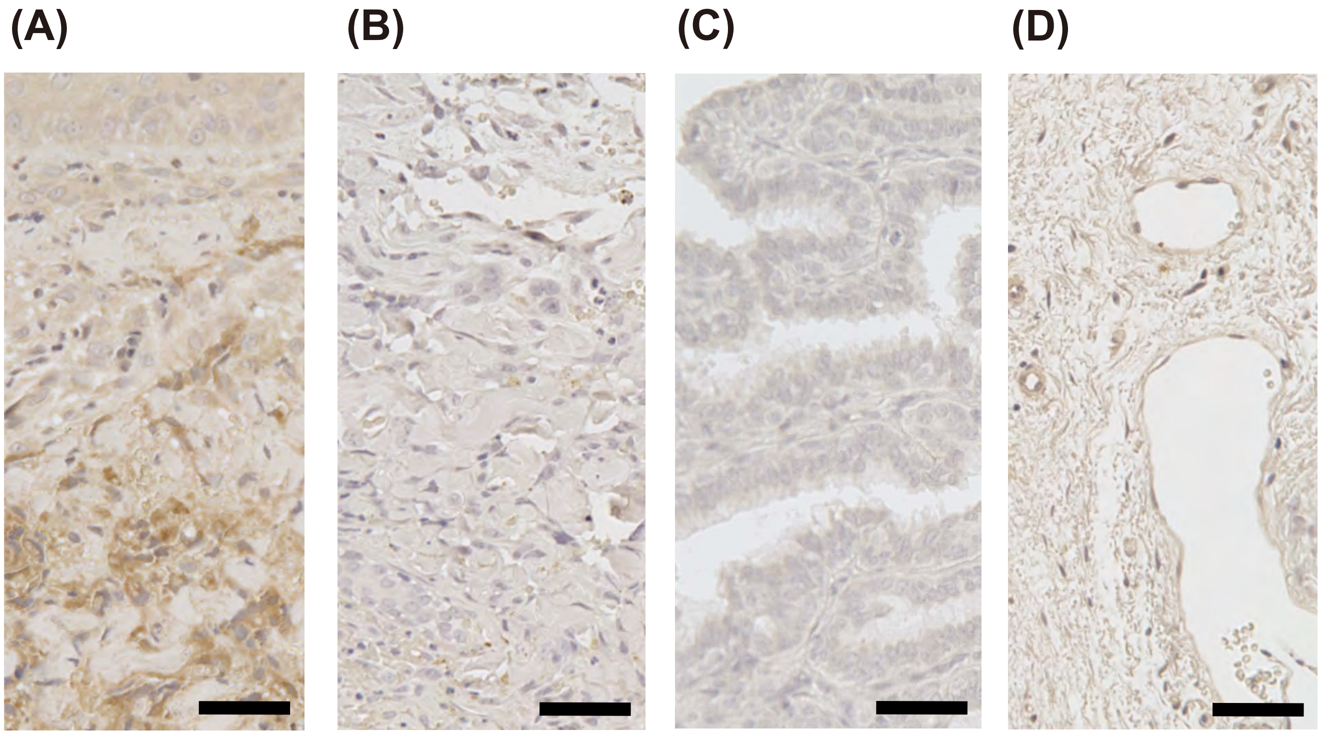

Supplementary Figure 1.

Representative IHC images showing IL-13Rα2 expression in angiosarcoma and control tissues.

(A) Angiosarcoma sample with strong IL-13Rα2 staining (IHC H-score = 270).

(B) Angiosarcoma sample with weak IL-13Rα2 staining (IHC H-score = 25).

(C) A representative sample hemangioma (IHC H-score = 10).

(D) A representative healthy skin sample (IHC H-score = 40).

Scale bars = 50μm.

These images exemplify the range of immunoreactivity observed across different tissue types and correspond to the quantitative data shown in Fig. 2(I).
