# Supplementary material for: IL-13/IL-13Rα2 axis promotes proliferation of angiosarcoma cells
Source: Sci Rep. 2025 Aug 25;15:31236. doi: 10.1038/s41598-025-15933-6 (PMC12379233; doi:10.1038/s41598-025-15933-6)
Supplement: Supplementary file 2 — Supplementary Information 2. [file 41598_2025_15933_MOESM2_ESM.pdf]

Supplementary information

Supplementary Figure 2

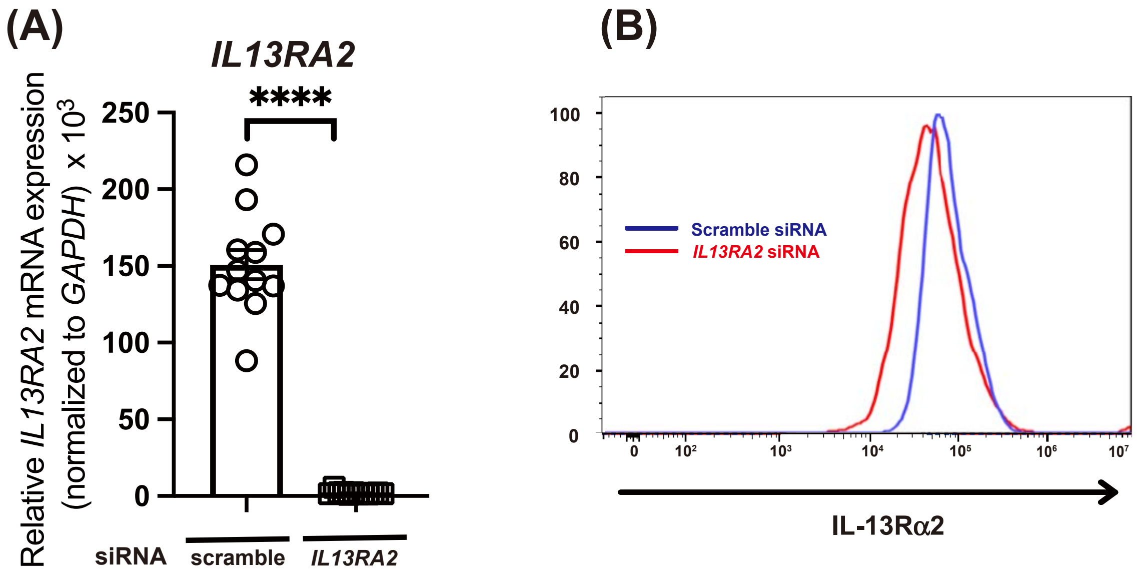

Supplementary Figure 2.

Knockdown efficiency of *IL13RA2* siRNA in MO-LAS-B cells.

(A) Relative mRNA expression of *IL13RA2* in MO-LAS-B cells transfected with scramble or *IL13RA2*-specific siRNAs, showing more than 95% knockdown efficiency in *IL13RA2* mRNA expression with specific siRNA (n = 12 for each group). The analysis confirmed >95% knockdown efficiency. ****p<0.0001.

(B) IL-13Rα2 expression was evaluated by flow cytometry 72 hours after transfection. IL-13Rα2 expression was reduced in siRNA-transfected cells, confirming knockdown efficiency.
