# Supplementary material for: IL-13/IL-13Rα2 axis promotes proliferation of angiosarcoma cells
Source: Sci Rep. 2025 Aug 25;15:31236. doi: 10.1038/s41598-025-15933-6 (PMC12379233; doi:10.1038/s41598-025-15933-6)
Supplement: Supplementary file 3 — Supplementary Information 3. [file 41598_2025_15933_MOESM3_ESM.pdf]

Supplementary information

Supplementary Table 1.
List of primers used for real-time PCR.

Human

Gene	Forward Primer (5’-3’)	Reverse Primer (5’-3’)
IL13RA2	GGAGCATACCTTTGGGACCTATTC	ATTGTCGGGTTTCATTTGTTGTTT
IL13RA1	GGGAAAAGGGAGGGAAAAGG	TGACACTGGGTTTGCTTAGGTATG
VEGFA	AAGGAGGAGGGCAGAATCAT	TGGTGATGTTGGACTCCTCA
GAPDH	CACCCACTCCTCCACCTTTG	CTCTCTTCCTCTTGTGCTCTTGCT
